# Hybrid ileal pouch with concomitant anti-refluxing and refluxing ureteroileal anastomosis

**DOI:** 10.1186/s12894-021-00828-1

**Published:** 2021-06-11

**Authors:** Se Young Choi, Bumjin Lim, Byung Hoon Chi, Jung Hoon Kim, Wonchul Lee, Dalsan You, Choung-Soo Kim

**Affiliations:** 1 Department of Urology, Chung-Ang University Hospital, Chung-Ang University College of Medicine, 102, Heukseok-ro, Dongjak-gu, Seoul, 06973 Korea; 2 Biomedical Research Institute, Chung-Ang University Hospital, 102, Heukseok-ro, Dongjak-gu, Seoul, 06973 Korea; 3 Department of Urology, Asan Medical Center, University of Ulsan College of Medicine, 88 Olympic-ro 43-gil, Songpa-gu, Seoul, 05505 Korea; 4 Department of Urology, Hanil General Hospital, Seoul, Korea

**Keywords:** Ileal pouch, Urinary diversion, Reflux, Neobladder, Renal function

## Abstract

**Purpose:**

We report our preliminary experience of using a hybrid ileal pouch, assessing oncologic outcomes, complications, voiding, and renal function.

**Methods:**

The study included 25 patients with bladder cancer treated with radical cystectomy with a hybrid ileal pouch with concomitant anti-refluxing and refluxing anastomosis, performed by a single surgeon. The patients were divided into two groups (first and last cases) according to the surgery date. Postoperative complications, separate renal function by renal scan, voiding function by uroflowmetry with residual urine, and oncologic outcomes were assessed.

**Results:**

The surgery duration was shorter in the last cases than the first cases. The voiding volume increased with time. There were 23 cases of grade 3 complication in 12 patients and one case of grade 4 complication (sepsis). In the first cases, ureterovesical stenosis occurred in five cases, whereas in the last cases, there were no cases of stenosis. In separate renal function, there was no difference between the left and right side or between the first and last cases.

**Conclusions:**

The hybrid ileal pouch showed acceptable oncologic and functional outcomes and complications; therefore, it can be used according to the appropriate surgical situation with a relatively short bowel segment during neobladder construction.

## Introduction

An orthotopic neobladder constructed following radical cystectomy is preferred by patients for physiologic and anatomical reasons. However, there are many complications with this reconstruction, including declining renal function or voiding difficulty, even with experienced surgeons [1, 2]. The ideal orthotopic neobladder preserves renal function and enables the anatomical storage and emptying of urine without an external appliance. The deterioration of renal function is one of the most important adverse events related to urinary diversion, as chronic kidney disease is associated with substantial cardiovascular morbidity and mortality [3]. There is little evidence regarding the impact on renal function of specific types of urinary diversion, that is, anti-refluxing or refluxing techniques. Some authors have suggested that the anti-refluxing type can reduce upper tract scarring by reducing vesicoureteral reflux [4]. Others have maintained that a low-pressure reservoir with a chimney helps to preserve renal function [5]. A recent randomized clinical trial comparing a T-pouch with an anti-refluxing mechanism versus a Studer pouch did not show significant differences in preservation of renal function at 3 years [6].

Upper tract recurrence following cystectomy has been reported in ~ 5% of cases [7]. Positive ureteral margin at cystectomy has been associated with upper tract recurrence, and the negative conversion may help to decrease the risk of recurrence [8]. The situation of a short remnant ureter occurs because of serial additional resection. The use of a hybrid ileal pouch was introduced to resolve the deficiency of the short ureter. We concerned the effect of anti-refluxing and refluxing ureteroileal anastomosis on separate renal function in the same condition of the neobladder. Here, we report our preliminary experience of radical cystectomy with a hybrid ileal pouch, assessing the oncologic outcomes, complications, voiding, and renal function following surgery.

## Materials and methods

The need for informed consent was waived by the Institutional Review Board of the Asan Medical Center owing to the minimal risk of harm. After receiving approval (2018–1041) from the institutional review board at the Asan Medical Center, all methods were carried out in accordance with relevant guidelines and regulations. We retrospectively reviewed the medical records of 25 patients who underwent radical cystectomy and hybrid ileal neobladder construction for bladder cancer between June 2009 and September 2018. In general, patients with favorable performance status and renal function were selected. Before surgery, clinical examination, a laboratory profile, computerized tomography, and a renal scan (^99m^Tc-diethylene triamine penta-acetic acid or ^99m^Tc-mercaptoacetyltriglycine), which show separate renal function, were evaluated. The baseline characteristics of patients and tumors and perioperative data including pathology and surgical technique were collected. The first cases (group A; n = 17) were performed between June 2009 and March 2010, and the last cases (group B; n = 8) were performed after May 2016.

**Fig. 1:**
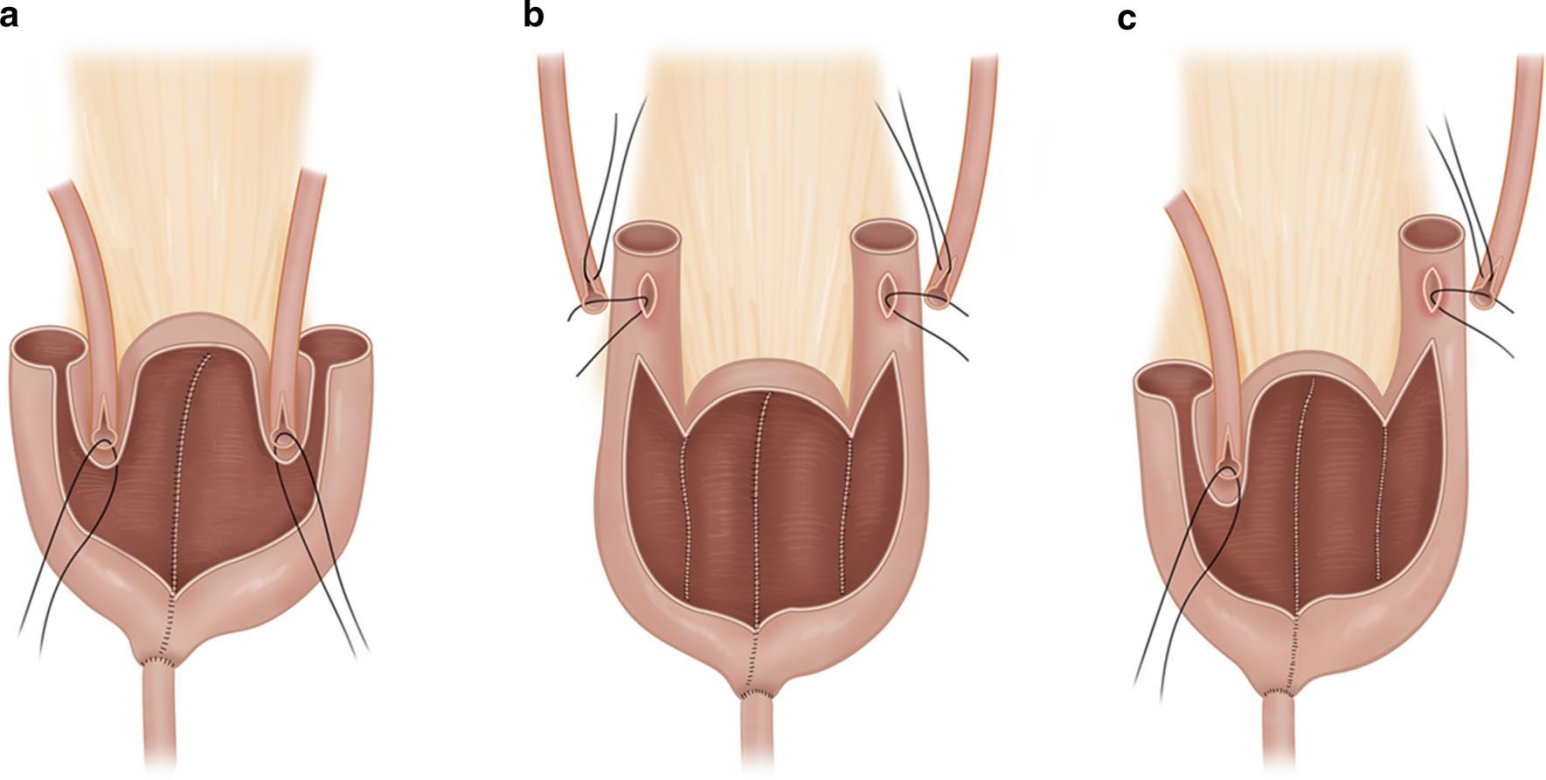
Concept of the hybrid ileal pouch that was composed of a Ghoneim pouch (anti-refluxing type, (**a**) and Hautmann pouch (refluxing type, **b**) on each side (**c**)

All patients had undergone radical cystectomy, urinary diversion, and lymph node dissection. The surgical procedures were performed by one experienced surgeon (C.-S. K.) who had 21.4 cases of the average annual surgeon volume for radical cystectomy over 10 years and had performed > 250 radical cystectomies as the operator before 2009. Between April 2010 and April 2016, the surgeon performed 117 cases of additional radical cystectomy. In general, for the neobladder, an ileal reservoir was constructed with ureteroileal reimplantation in extramural tunnels to prevent reflux (Ghoneim pouch, Fig. 1a) which can be made with a short length of bowel segment ~ 40–50 cm [2]. The most common reason to make a hybrid pouch was due to shortening of the remnant ureter due to additional resection following positive intraoperative frozen section. The surgeon modified the Ghoneim pouch with a Hautmann pouch (Fig. 1b) [9] and constructed the hybrid pouch (Fig. 1c). A 45–60 cm length of ileum was used for the neobladder, which included 6–10 cm of one chimney. A 1-cm-sized hole was made in the dependent lesion of the neobladder for urethra anastomosis. One ureter was connected to the chimney directly by the refluxing technique, and the other ureter was implanted into the reentrant lesion of the neobladder using the anti-refluxing extramural tunneling technique. In cases of a left-side chimney, the chimney was overlaid on the colon. Ureteral stents were inserted on both sides, and suprapubic cystostomy was maintained until self-voiding following cystography.

Renal function was assessed using the estimated glomerular filtration rate (GFR) calculated with the Chronic Kidney Disease Epidemiology Collaboration equation [10]. We evaluated the separate GFR for each kidney using a renal nucleotide scan. Following radical cystectomy, the patients were generally followed up every 3 months during the first year, every 6 months during years 2–6, and annually thereafter. History taking, physical examination, blood laboratory investigations, urine sedimentation, culture, cytology, renal scan, uroflowmetry with residual urine, and computerized tomography were evaluated. The median duration from the date of surgery to the final follow-up date was 29.8 months (interquartile range [IQR] 18.5–109.3). The follow-up duration of group A and B were 58.1 (IQR 20.8–123.9) months and 25.2 (IQR 14.9–38.1) months (*P* = 0.086).

The clinicopathologic characteristics were compared among the three groups using Fisher's exact test for categorical variables and a t-test or Mann Whitney *U* test for continuous variables according to normality by the Kolmogorov–Smirnov test. The characteristics were described as the mean ± standard deviation with IQR or numbers with percentages. Cox proportional-hazard regression was used to compare recurrence and overall survival between groups. All statistical analyses were performed using IBM SPSS Statistics Version 21 (IBM Corporation, Somers, NY, USA) and R version 3.4.3 (R Project for Statistical Computing; http://www.r-project.org/). All *P* values were two-tailed, and *P* < 0.05 was considered significant.

## Results

The clinical and pathological characteristics of the group A and B are presented in Table 1. There were no differences in baseline characteristics between the group A and B. In the entire cohort, the mean age was 62.3 ± 9.3 years (IQR 57.5–67.0). The majority of patients (88.0%) were male, and 48.0% had a clinical T2 or stage of lower. Among the cohort, 4 patients (16.0%) had clinical lymph nodes and 19 patients (76.0%) had a grade 3 tumor that was obtained at transurethral resection of the bladder tumor. Neoadjuvant chemotherapy was administered to three patients (12.0%).

**Table 1:** Clinical and pathological characteristics

	Group A (N = 17)	Group B (N = 8)	Total (N = 25)	*p*
Age (years)	63.4 ± 7.8 [59.0; 67.0]	60.0 ± 12.0 [50.5; 69.0]	62.3 ± 9.3 [57.5; 67.0]	0.410
Male gender	15 (88.2%)	7 (87.5%)	22 (88.0%)	1.000
Height	165.0 ± 7.9 [161.0; 170.8]	165.5 ± 4.9 [162.7; 169.6]	165.2 ± 7.0 [160.1; 169.7]	0.887
Weight	66.6 ± 8.7 [60.2; 75.3]	64.7 ± 12.8 [56.8; 76.8]	66.0 ± 9.9 [60.2; 76.0]	0.665
Body mass index (kg/m^2^)	24.5 ± 3.5 [21.6; 26.4]	22.8 ± 3.5 [20.5; 26.4]	24.0 ± 3.5 [21.3; 26.4]	0.293
Diabetes mellitus	3 (17.6%)	2 (25.0%)	5 (20.0%)	1.000
Hypertension	6 (35.3%)	2 (25.0%)	8 (32.0%)	0.956
*Clinical T stage*				0.571
≤ T2	7 (41.2%)	5 (62.5%)	12 (48.0%)	
≥ T3	10 (58.8%)	3 (37.5%)	13 (52.0%)	
Clinical lymph node positive	3 (17.6%)	1 (12.5%)	4 (16.0%)	1.000
*Preoperative grade*				0.544
G2	3 (20.0%)	0 (0.0%)	3 (12.0%)	
G3	12 (80.0%)	7 (100.0%)	19 (76.0%)	
Neoadjuvant chemotherapy	1 (5.9%)	2 (25.0%)	3 (12.0%)	0.476

Mean ± standard deviation; []: Interquartile range

The duration of surgery was shorter in the group B (389.9 ± 52.8 min) than the group A (499.4 ± 61.7 min, *P* < 0.001) (Table 2). There was no difference in hospitalization time (*P* = 0.092), T (*P* = 0.154), and N stage (*P* = 0.439). The majority (88.2%) of group A underwent super-extended lymphadenectomy up to the inferior mesenteric artery, whereas all of the group B had extended lymphadenectomy up to the great vessel bifurcation. In almost all cases, the chimney was on the left side (96.0%). In terms of pathologic T stage, 52.0% of cases were T2 or lower, and 24.0% were N1 or higher. We removed an average of 31.3 ± 13.0 lymph nodes. There was one positive soft tissue margin. During follow-up periods, 9 patients (36.0%) had recurrences and 14 patients (56.0%) succumbed to mortality. There was no upper track recurrence. In Cox analysis, group was not a significant factor in recurrence (hazard ratio [HR] 2.049, 95% confidence interval [CI] 0.548–7.664, p = 0.286) and overall survival (HR 4.023, 95% CI 0.934–17.329, *P* = 0.062).

**Table 2:** Perioperative characteristics, pathology, and oncologic outcome

	Group A (N = 17)	Group B (N = 8)	Total (N = 25)	*p*
Operation time (min)	499.4 ± 61.7 [464.0; 531.0]	389.9 ± 52.8 [364.5; 424.0]	464.4 ± 77.9 [420.5; 510.0]	** < 0.001**
Hospitalization time (days)	54.8 ± 52.6 [32.0; 49.0]	32.1 ± 14.5 [23.5; 33.0]	48.2 ± 45.7 [26.0; 49.0]	0.092
*Chimney side*				0.694
Left	17 (100.0%)	7 (87.5%)	24 (96.0%)	
Right	0 (0.0%)	1 (12.5%)	1 (4.0%)	
*Pelvic lymph node dissection*				** < 0.001**
Standard	1 (5.9%)	0 (0.0%)	1 (4.0%)	
Extended	1 (5.9%)	8 (100.0%)	9 (36.0%)	
Super-extended	15 (88.2%)	0 (0.0%)	15 (60.0%)	
*Pathologic T stage*				0.154
≤ T2	11 (64.7%)	2 (25.0%)	13 (52.0%)	
≥ T3	6 (35.3%)	6 (75.0%)	12 (48.0%)	
*Pathologic N stage*				0.439
0	14 (82.4%)	5 (62.5%)	19 (76.0%)	
1	1 (5.9%)	0 (0.0%)	1 (4.0%)	
2	1 (5.9%)	1 (12.5%)	2 (8.0%)	
3	1 (5.9%)	2 (25.0%)	3 (12.0%)	
Removed lymph node count	32.6 ± 14.0 [23.0; 39.0]	28.5 ± 10.8 [21.5; 39.0]	31.3 ± 13.0 [22.0; 39.0]	0.475
Number of positive lymph node	0.3 ± 0.7 [0.0; 0.0]	1.4 ± 2.7 [0.0;2.0]	0.6 ± 1.6 [0.0;0.0]	0.532
*Postoperative grade*				0.513
G2	3 (18.8%)	0 (0.0%)	3 (12.5%)	
G3	13 (81.2%)	8 (100.0%)	21 (87.5%)	
Positive soft margin	0 (0.0%)	1 (12.5%)	1 (4.0%)	0.694
Lymphovascular invasion	3 (17.6%)	4 (50.0%)	7 (28.0%)	0.229
Adjuvant chemotherapy	1 (5.9%)	1 (12.5%)	2 (8.0%)	1.000
Recurrence	5 (29.4%)	4 (50.0%)	9 (36.0%)	0.580
Death	10 (58.8%)	4 (50.0%)	14 (56.0%)	1.000

Bold values indicate statistical significance at* P* < 0.05

Mean ± standard deviation; []: Interquartile range

**Fig. 2:**
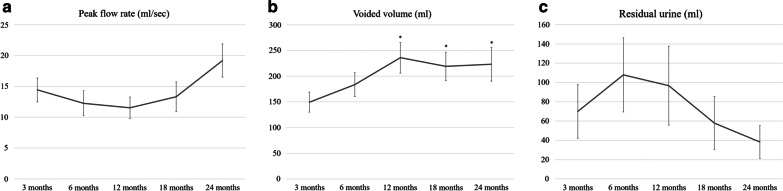
Peak flow rate (**a**) and voided volume (**b**) of uroflowmetry and residual urine (**c**) following radical cystectomy with a hybrid ileal pouch

**Fig. 3:**
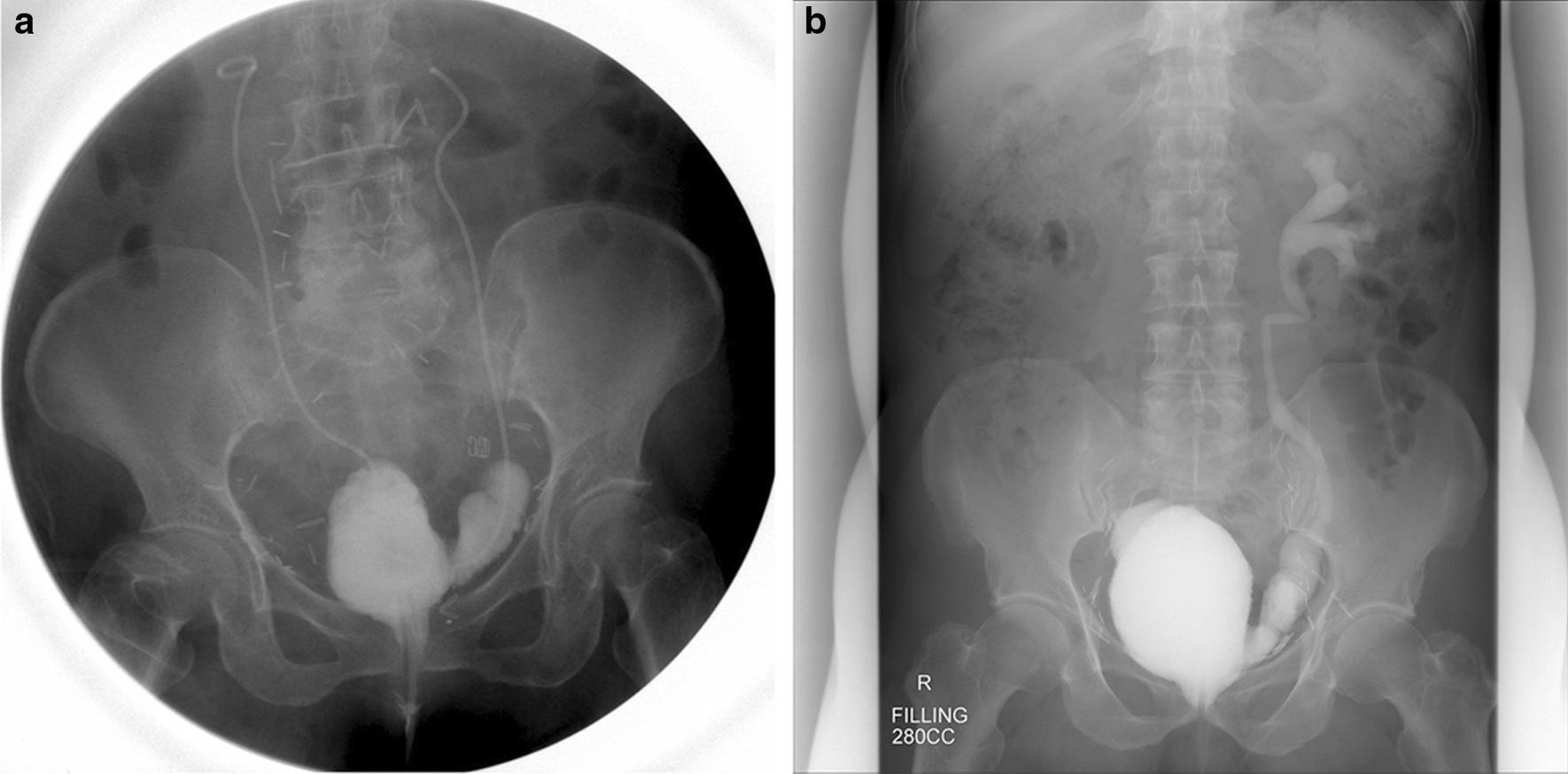
Representative images of hybrid ileal pouch. Cystography at 2 weeks post-surgery (**a**) and voiding cystourethrography 1 year post-surgery (**b**). *P < 0.05 compared with value at 3 months

There were no cases in which to keep Foley catheter or clean intermittent catheter. The trends of voiding values of uroflowmetry and residual urine are shown in Fig. 2. There was no statistical difference among the values of peak flow rate and residual urine; however, voiding volume increased from 149.4 ± 19.5 (3 months) to 235.9 ± 29.9 (12 months, *P* = 0.016). The forms of the hybrid ileal pouch at 2 weeks and 1 year post-surgery are shown in Fig. 3. At 2 weeks, ureteral stents were in place on both sides. At 1 year, the pouch appeared larger than that at 2 weeks. Vesicoureteral reflux was observed on the chimney side, but there was no reflux in the anti-refluxing side.

Table 3 shows the complications recorded. A total of 50 complications were reported in 17 patients (68.0%). There were 30 complications during the admission period and 20 complications following discharge. Almost all complications were Clavien grade 2 or 3. There were 23 cases of grade 3 in 12 patients (48.0%) and 1 case of grade 4, which was sepsis requiring management in an intensive care unit. Urethrovesical anastomosis leakage was the most common complication. In the group A, there were three cases of left ureterovesical stenosis and two cases of right ureterovesical stenosis. No ureterovesical stenosis was reported in the group B. In the patients with the stenosis, the ipsilateral GFR of the stenosis was significantly decreased (pre-surgery: 40.2 ± 6.9, 1 year: 18.6 ± 10.4, 2 year: 17.0 ± 12.3).

**Table 3:** Postoperative complication during follow-up period

	Group A (N = 17)	Group B (N = 8)	Total (N = 25)	Grade 3/4
*Short-term complications (during admission)*				
Urethrovesical anastomosis leakage	7 (41.2%)	2 (28.6%)	8 (32.0%)	1 (4.0%)
Wound dehiscence	3 (17.6%)	1 (14.3%)	4 (16.0%)	4 (16.0%)
Febrile infection	3 (17.6%)	0 (0.0%)	3 (12.0%)	0 (0.0%)
Ileus	2 (11.8%)	1 (14.3%)	3 (12.0%)	0 (0.0%)
Ureterovesical anastomosis leakage	2 (11.8%)	0 (0.0%)	2 (8.0%)	2 (8.0%)
Neobladder perforation	2 (11.8%)	0 (0.0%)	2 (8.0%)	2 (8.0%)
Abscess	2 (11.8%)	0 (0.0%)	2 (8.0%)	2 (8.0%)
Lymphocele	1 (5.9%)	1 (12.5%)	2 (8.0%)	2 (8.0%)
Ureterovesical stenosis (both)	1 (5.9%)	0 (0.0%)	1 (4.0%)	1 (4.0%)
Urethra stricture	0 (0.0%)	1 (14.3%)	1 (4.0%)	1 (4.0%)
Ileoileal anastomosis perforation	1 (5.9%)	0 (0.0%)	1 (4.0%)	1 (4.0%)
Sepsis	1 (5.9%)	0 (0.0%)	1 (4.0%)	1 (4.0%)
*Long-term complications (after discharge)*				
Ureterovesical stenosis	5 (29.4%)	0 (0.0%)	5 (20.0%)	5 (20.0%)
Left	3 (17.6%)	0 (0.0%)	3 (12.0%)	
Right	2 (11.8%)	0 (0.0%)	2 (8.0%)	
Fistula	4 (23.5%)	0 (0.0%)	4 (16.0%)	4 (16.0%)
Febrile infection	2 (11.8%)	2 (28.6%)	4 (16.0%)	0 (0.0%)
Small bowel obstruction	0 (0.0%)	1 (14.3%)	1 (4.0%)	1 (4.0%)
Abscess	1 (5.9%)	0 (0.0%)	1 (4.0%)	1 (4.0%)

**Fig. 4:**
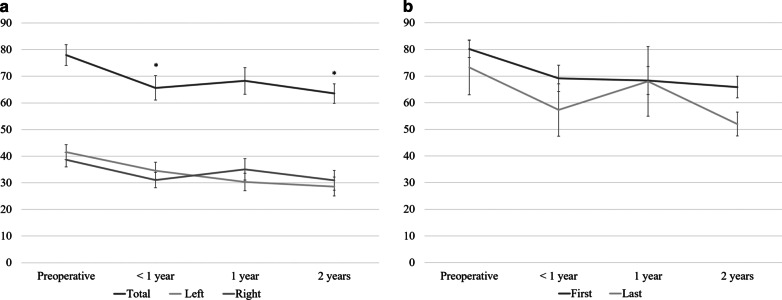
Trends in total glomerular filtration rate of each renal unit (**a**) and first and last cases (**b**). There was no difference between the left and right sides, or between the first (group A) and last cases (group B). *P < 0.05 compared with the mean preoperative value

The trends in GFR from pre-surgery to 2 years post-surgery are shown in Fig. 4. The total GFR decreased significantly from pre-surgery (78.0 ± 19.5) to 2 years post-surgery (63.6 ± 15.5, *P* = 0.013). There was no difference between the left and right sides or between the group A and B (*P* > 0.05).

## Discussion

Our preliminary results of radical cystectomy with a hybrid ileal pouch showed acceptable oncologic, complication and functional outcomes. There was no upper track recurrence among 24.0% of total recurrences. The recurrence rate can be confounded by the tumor stage. The 5 year recurrence-free survival rate has been reported as 62–68% and upper tract recurrence rates as 2.4–17% [11]. Upper track recurrence can be caused by both metachronous occurrence and intraluminal seeding [12]. Palou et al. reported that vesicoureteral reflux can increase the risk of upper track recurrence following transurethral resection [13]. In cases of cystectomy, the association between the type of ureteral anastomosis and upper track recurrence is unclear. As recurrence is rare and theoretically no tumor cell is present in the neobladder, urethral recurrence may be associated with the type of diversion between continence and incontinence (5% vs 9%) [14]. However, retrospective cases of urethral or prostate involvement would be avoided in designing a continent-type neobladder; thus, selection bias may be present [14]. Until now, there is no evidence that the neobladder type can increase the urethral recurrence risk. In our study, there was only one case (4%) of urethral recurrence.

In our study, the mortality rate was 56.0%. There were no cases of mortality related to the surgery itself. There was one case of sepsis (grade 4) that was caused by peritonitis following surgery; however, the patient was discharged (hospitalization 103 days) following recovery and survived during the 58 month period without recurrence. In a previous study of 11,933 patients who underwent radical cystectomy, short-term mortality rates were similar between neobladder and ileal conduit after propensity when matching by age, race, gender, stage, facility type, histology, and comorbidity [15].

The complication rate following neobladder reconstruction may be associated with the experience of the surgeon, method of surgery, or condition of the patients. The method of data collection may also influence the assessment of complications. In our study, grade 3 or higher complications were present in 12 patients (48.0%). This rate was higher than reported by others. Hautmann et al. reported an incidence of 21.8% grade 3 or higher complications [9], and Ali-El-Dein et al. reported a 35% incidence of late complications [16]. However, the initial technique may lead to more complications. Gakis et al. reported a 39.2% 90 day complication rate as initial results [17]. Our results were from not only the initial 90 days but also all follow-up periods. In addition, the group B had much fewer complications than the group A. In particular, ureterovesical stenosis decreased from five cases to zero. In the group B, the hospitalization stay was similar to other studies [18]. This may be associated with the modified ureter-fixation technique, with one point of the distal region of the spatulated ureter sutured with full depth from mucosa to serosa, but the other regions of the remnant ureter were sutured on only the mucosa between the ureter and ileum. The anti-refluxing extramural tunnel region should also be sutured on the mucosa only. Although only mucosa was sutured on the regions, there was no ureterovesical anastomosis leakage or neobladder perforation in the group B. In addition, the connection of the mid region of the hybrid pouch to the opened chimney lumen should be positioned in the upper level to the opened chimney prior to closure of the neobladder for sparing chimney lumen with less tension. The upfront chimney beyond the rectum can assist in ensuring the ureter is not angulated or under tension. Although the patients with the ureterovesical stenosis underwent percutaneous nephrostomy or ureteral stent, their ipsilateral GFR was significantly decreased. Therefore, the above techniques about mucosa-to-mucosa and tension-free are important to prevent the stenosis. At last, group B showed relatively acceptable data about complications maintaining renal functions.

The voiding function was maintained reasonably well. At 1 year post-surgery, the void volume reached at least 200 mL. The hybrid pouch was formed into a spherical shape; this spherical shape resembling the original bladder shape may have advantages including constant pressure stresses on the entire wall and prevention of internal folding of the walls. Therefore, the spherical shape may be helpful to voiding function.

We compared the separate renal function of each side. The anti-refluxing types were mainly on the right side, and refluxing types were mainly on the left side. The separate renal function did not differ significantly between the right and left sides. At 2 years, ~ 81.5% of total GFR was preserved. The decreases of GFR are commonly observed following radical cystectomy, and the risk factors include hydronephrosis, pyelonephritis, and ureterovesical stricture [19]. Harraz et al. also reported GFR decreases of ~ 10% after 2 years, with no difference between the refluxing and anti-refluxing type [20]. To establish an ideal pouch to protect renal function, further investigations are required.

This study had some limitations. As a retrospective study, there may have selection bias, including a short remnant ureter. The extent of node dissection was different between 2 groups, because we found the survival gain from super-extended lymphadenectomy was limited [21]. The technique of lymphadenectomy might not be associated with the rates of ureteroileal stricture, because of far region from the lymph node fields. There is also the possibility of the under-reporting of complications; in particular, the presence or degree of incontinence was not surveyed. However, we periodically checked uroflowmetry with residual urine. The small sample size and short follow-up requires additional investigation. In addition, this preliminary technique needs to be established consistently. The comparison of various pouches is difficult because of various patient or tumor characteristics and various surgical techniques. This study can provide clues in distinguishing between anti-refluxing and refluxing types. Use of a hybrid ileal pouch may have advantages for a short remnant ureter, owing to the relatively short length of bowel segment required and anti-refluxing mechanism to prevent direct refluxing infection.

## Conclusions

The hybrid ileal pouch had concomitant characteristics of anti-refluxing and refluxing ureteroileal anastomosis. There were no differences in the GFR of each side or in ureterovesical stenosis between the anti-refluxing and refluxing anastomosis side. Skilled techniques may reduce significant complications according to the accumulation of experiences. Our preliminary data of use of a hybrid ileal pouch showed acceptable complication, oncologic, and functional outcomes in patients with bladder cancer. Therefore, the hybrid ileal pouch can be applied according to the appropriate surgical situation with a relatively short bowel segment during neobladder surgery.

## Data Availability

The datasets used and/or analysed during the current study available from the corresponding author on reasonable request.

## Abbreviations

GFR: Glomerular filtration rate
IQR: interquartile range
